# Application of the Expert Recommendations for Implementing Change (ERIC) compilation of strategies to health intervention implementation in low- and middle-income countries: a systematic review

**DOI:** 10.1186/s13012-023-01310-2

**Published:** 2023-10-30

**Authors:** Kathryn L. Lovero, Christopher G. Kemp, Bradley H. Wagenaar, Ali Giusto, M. Claire Greene, Byron J. Powell, Enola K. Proctor

**Affiliations:** 1 Department of Sociomedical Sciences, Columbia University Mailman School of Public Health, New York, NY USA; 2 Department of International Health, Johns Hopkins Bloomberg School of Public Health, Baltimore, MD USA; 3 Department of Global Health, University of Washington, Seattle, WA USA; 4 Department of Epidemiology, University of Washington, Seattle, WA USA; 5 Department of Psychiatry, Columbia University Irving Medical Center, New York State Psychiatric Institute, New York, NY USA; 6 Program On Forced Migration and Health, Heilbrunn Department of Population and Family Health, Columbia University Mailman School of Public Health, New York, NY USA; 7 Brown School, Center for Mental Health Services Research, Washington University in St. Louis, St. Louis, MO USA; 8 Center for Dissemination & Implementation, Institute for Public Health, Washington University in St. Louis, St. Louis, MO USA; 9 Division of Infectious Diseases, John T. Milliken Department of Medicine, School of Medicine, Washington University in St. Louis, St. Louis, MO USA

**Keywords:** Implementation strategy, Strategy specification, Systematic review, Global health

## Abstract

**Background:**

The Expert Recommendations for Implementing Change (ERIC) project developed a compilation of implementation strategies that are intended to standardize reporting and evaluation. Little is known about the application of ERIC in low- and middle-income countries (LMICs). We systematically reviewed the literature on the use and specification of ERIC strategies for health intervention implementation in LMICs to identify gaps and inform future research.

**Methods:**

We searched peer-reviewed articles published through March 2023 in any language that (1) were conducted in an LMIC and (2) cited seminal ERIC articles or (3) mentioned ERIC in the title or abstract. Two co-authors independently screened all titles, abstracts, and full-text articles, then abstracted study, intervention, and implementation strategy characteristics of included studies.

**Results:**

The final sample included 60 studies describing research from all world regions, with over 30% published in the final year of our review period. Most studies took place in healthcare settings (*n* = 52, 86.7%), while 11 (18.2%) took place in community settings and four (6.7%) at the policy level. Across studies, 548 distinct implementation strategies were identified with a median of six strategies (range 1–46 strategies) included in each study. Most studies (*n* = 32, 53.3%) explicitly matched implementation strategies used for the ERIC compilation. Among those that did, 64 (87.3%) of the 73 ERIC strategies were represented. Many of the strategies not cited included those that target systems- or policy-level barriers. Nearly 85% of strategies included some component of strategy specification, though most only included specification of their action (75.2%), actor (57.3%), and action target (60.8%). A minority of studies employed randomized trials or high-quality quasi-experimental designs; only one study evaluated implementation strategy effectiveness.

**Conclusions:**

While ERIC use in LMICs is rapidly growing, its application has not been consistent nor commonly used to test strategy effectiveness. Research in LMICs must better specify strategies and evaluate their impact on outcomes. Moreover, strategies that are tested need to be better specified, so they may be compared across contexts. Finally, strategies targeting policy-, systems-, and community-level determinants should be further explored.

**Trial registration:**

PROSPERO, CRD42021268374.

**Supplementary Information:**

The online version contains supplementary material available at 10.1186/s13012-023-01310-2.

Contributions to the literature

- The ERIC compilation of implementation strategies has been widely adopted in high-income settings, but its usage and relevance across low- and middle-income countries (LMICs) have not been systematically explored.
- This systematic review demonstrates that ERIC use is increasing in LMICs. Most individual ERIC strategies have been applied, though few targeted organizational- and policy-level change.
- ERIC application was inconsistent and the specification of strategies was low; only one study tested strategy effectiveness.
- Findings point to a need for training and resources to support specification and testing of implementation strategies in LMICs to build the evidence base on implementation strategy effectiveness across diverse settings.

## Background

The past two decades have been marked by rapid growth in the field of implementation science to address the large research-to-practice gap across contexts and health areas [1]. In recent years, the field’s focus has shifted from defining barriers and facilitators of implementing evidence-based practices to identifying strategies that effectively address and overcome these barriers. Implementation strategies are generally defined as the approaches or techniques used to enhance the adoption, implementation, sustainment, and scale-up of an evidence-based practice [2, 3]. These strategies vary in complexity and can target determinants at the intervention-, patient-, provider-, organization-, community-, policy-, and funding levels [4, 5].

Though the evidence base on implementation strategies is growing, current data on strategy effectiveness is mixed, with high variation in strategy effects observed across studies and outcomes [6–11]. Several reasons may contribute to this variation. It may be that certain strategies are not sufficient to improve implementation outcomes across contexts; it may also be that strategies were not appropriately matched to the contextual determinants or tailored to the setting [12]. However, reporting on implementation strategies often lacks the necessary information to determine why a strategy was or was not effective; for example, information on how a strategy was selected, adapted, and operationalized and whether or not the strategy was carried out as intended [7, 13–17]. As such, calls for consistent, detailed reporting of implementation strategies have emerged in tandem with calls for increased research on strategy effectiveness [2, 3, 18–20].

To aid reporting efforts, the field has developed taxonomies of implementation strategies [21, 22] and a methodology for specifying implementation strategies [3]. One strategy taxonomy, the Expert Recommendations for Implementing Change (ERIC) project, built upon a narrative review of the literature [23] and used a modified Delphi process to develop a compilation of implementation strategies, comprising 73 discrete strategies [21] which can be further grouped into nine thematic clusters [24]. The ERIC compilation has become the most commonly used taxonomy of strategies in the field of implementation science, with over 3000 citations. It has enabled a standardized language for naming implementation strategies that have been used to characterize implementation efforts both in prospective and retrospective analyses [25–30]. To support standardized strategy specification, Proctor et al. [3] developed guidelines to help stakeholders operationalize strategies based on specific domains, including the strategy actor, action, action target, temporality, dose, implementation outcome affected, and justification. These specification guidelines are consistent with the Patient Centered Outcomes Research Institute's Standards for Studies of Complex Interventions [31], and have the potential to not only improve our understanding of implementation strategy mechanisms, but also the required parameters for replication in other research and practice settings.

In low- and middle-income countries (LMICs), implementation science has become a key tool for helping to bridge the research-to-practice gap that is larger than that of high-income countries [32, 33]. As such, the need to identify effective implementation strategies for efficient, effective delivery of evidence-based practices is imperative in these settings. In recent years, formal implementation research in LMICs has expanded as funding sources increasingly recognize the utility of this work. There is now a growing evidence base for using implementation frameworks [34, 35] and measures [36, 37], and specific determinants to implementation [38] in LMICs have been identified. However, little is known about effective implementation strategies in these settings [39].

The purpose of the present study is to report on the application of the ERIC compilation of implementation strategies in LMICs and provide recommendations to the field for improving its application moving forward. Our aims are twofold: (i) to systematically review the literature on the use of ERIC strategies in LMICs, including which specific strategies have been included in the research, how they were selected, how the strategies were used (i.e., specification and targeted intervention/health condition/population), and how they were adapted; and (ii) to assess evidence for the effectiveness of specific ERIC implementation strategies in LMICs.

## Methods

We registered our systematic review protocol in the International Prospective Register of Systematic Reviews (PROSPERO # CRD42021268374) and followed the Preferred Reporting Items for Systematic Reviews and Meta-Analyses (PRISMA) guidelines [40]. See Additional file 1 for the completed PRISMA checklist.

### Search strategy

We searched PubMed, CINAHL, PsycINFO, CINAHL, EMBASE, SCOPUS, and Web of Science until March 27, 2023, to identify original peer-reviewed research in any language that cited either (1) the original implementation strategies compilation paper [23], (2) the ERIC compilation [21], or (3) the strategy categorization [24], or that mentioned the ‘Expert Recommendations for Implementing Change’ or ‘ERIC’ in the title or abstract, and that took place within an LMIC. LMIC classification was determined based on World Bank criteria [41]. The full search strategy for all databases is presented in Additional file 2.

### Study selection

Covidence was used to remove duplicate studies and to conduct study screening [42]. A mix of two authors from a team of five (KL, CK, CG, AG, and BW) independently screened all titles, abstracts, and full-text articles, and noted reasons for excluding studies during full-text review. Studies passed the title/abstract screening stage if the title or abstract referenced the implementation of a health-related intervention and if it was possible that the study had been conducted in an LMIC. Studies passed the full-text screening stage if all criteria above were met and the study described the use of the ERIC compilation in implementation strategy selection, development, or classification (i.e., manuscripts that cited ERIC—for example, in the introduction or discussion—without indicating the application to the study strategies were excluded). Discrepancies in eligibility assessments were resolved through discussion until a consensus was reached.

### Data abstraction

Five authors (KL, CK, CG, AG, and BW) independently piloted a structured abstraction form with two studies each using a shared Google Sheets spreadsheet; all co-authors reviewed, critiqued, and approved the form. One of the two authors (CK and KL) then abstracted the study, intervention, and implementation strategy characteristics for the remaining studies (Additional file 3), while the other author-verified each abstraction, and then resolved any disagreement through discussion.

At the study level, we abstracted study settings, objectives, study design, and methods, whether the manuscript reported a study protocol or study results, any implementation research frameworks used, years of data collection, study populations, implementation outcomes reported, patient health and other outcomes reported, study limitations, and conclusions or lessons learned. We noted the types of independent variables represented in each study (i.e., intervention, implementation strategy, or context) based on which were systematically varied. Within each study, we also collected intervention names, intervention descriptions, associated health conditions, and target populations.

We then abstracted the discrete implementation strategies used and described in each study. At the implementation strategy level, we included descriptions of each strategy and noted whether the strategies were explicitly mapped to the ERIC strategy compilation, or to other strategy taxonomies (e.g., Behavior Change Wheel) [43]. We then abstracted each of the components of implementation strategy specification [3]; actor, action, action target, temporality, dose, implementation outcome(s) affected, and justification. We also noted any description of the hypothesized mechanism of action [19, 44], any description of adaptations to the implementation strategy [18, 45], and any assessment of implementation strategy fidelity [46]. Finally, we noted whether implementation strategy effect estimates were reported. Risk of bias was not assessed, as only one study evaluated strategy effectiveness and thus no meta-analysis of effectiveness was conducted.

### Analysis

Percentages were calculated for all categorical variables; these were used to summarize study, intervention, and implementation strategy characteristics. Quantitative meta-analysis of study findings was not possible given the heterogeneity in research questions and outcomes as well as the insufficient numbers of studies evaluating implementation strategy effects.

## Results

**Fig. 1:**
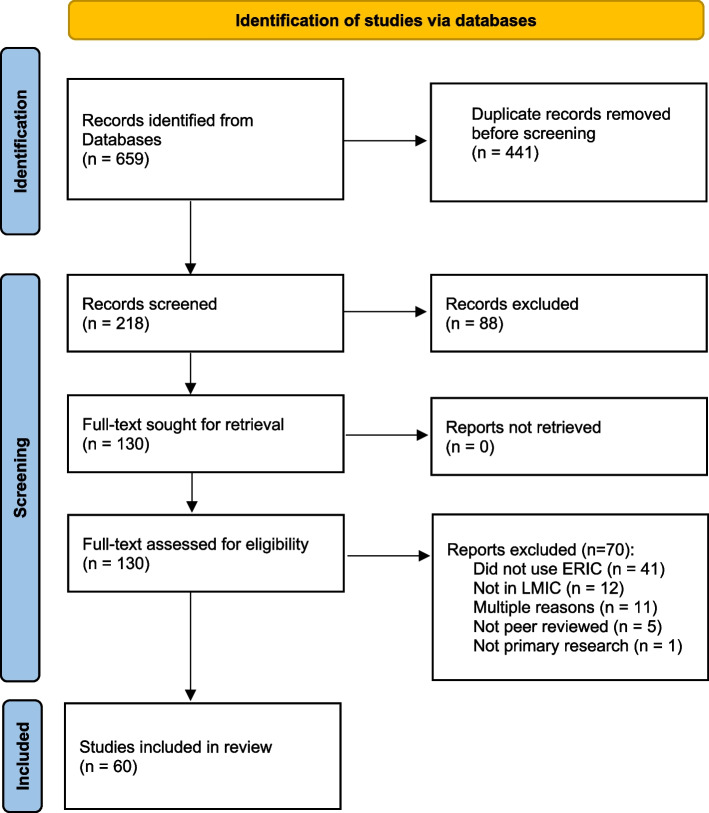
PRISMA 2020 flowchart of systematic review

The database search yielded 659 articles, of which 441 were duplicates. We screened the remaining 218 article titles and abstracts and excluded 88, leaving 130 for full-text review. Of these, 41 were excluded that did not use ERIC (i.e., only cited ERIC in the manuscript introduction or discussion, without application to the present study), 12 that did not take place in an LMIC, 11 that did not meet multiple inclusion criteria, 5 that were not peer-reviewed, and 1 that was not primary research (Fig. 1).

The final sample included 60 studies (Table 1, see Additional file 3 for individual study characteristics), all published in English. The first study using ERIC in an LMIC was published in 2016, and the number of studies using ERIC in LMICs increased over the years, from just 1 in 2016 to 20 in 2022. Studies included data collected in all six WHO Regions, with the majority being conducted in the African region (*n* = 36, 60.0%). Most studies focused on healthcare settings (*n* = 52, 86.7%), while just 11 (18.3%) focused on community and four (6.7%) on policy-level settings. The most common health conditions targeted in studies were infectious diseases (*n* = 19, 31.7%), maternal and child health (*n* = 10, 16.7%), and mental health and substance use (*n* = 10, 16.7%). Two studies did not focus on a specific health condition, but rather on strategies for implementing clinical trial recruitment and building a national framework for research.

**Table 1:** Study-level descriptive statistics (*n* = 60)

	*N* (%)
Year published	
2016	1 (1.8)
2017	2 (3.5)
2018	3 (5.3)
2019	2 (3.5)
2020	11 (19.3)
2021	16 (28.1)
2022	20 (35.1)
2023	2 (3.5)
WHO region^a^	
African	36 (60.0)
Western Pacific	7 (11.7)
South-East Asian	7 (11.7)
Americas	9 (15.0)
Eastern Mediterranean	2 (3.3)
European	2 (3.3)
Study setting^a^	
Healthcare	52 (86.7)
Community	11 (18.3)
Policy	4 (6.7)
Target health condition	
Cancer	1 (1.7)
Chronic non-communicable disease	7 (11.7)
General	9 (15.0)
Infectious disease	19 (31.7)
Maternal and child health	10 (16.7)
Mental health and substance use	10 (16.7)
None	2 (3.3)
Sexual and reproductive health	2 (3.3)
Study type	
Protocol	17 (28.3)
Empirical	43 (71.7)
Study population^a^	
Patients	31 (51.7)
Providers	43 (71.7)
Policymakers	14 (23.3)
Community members	8 (13.3)
Researchers	6 (10.0)
Process evaluation or formative study design^a^	
Formative implementation strategy design	17 (28.3)
Formative strategy design and prospective process evaluation	2 (3.3)
Prospective process evaluation	20 (33.3)
Retrospective strategy specification	9 (15.0)
Retrospective process evaluation	3 (5.0)
None	9 (15.0)
Impact evaluation study design	
Cluster RCT	9 (15.0)
Individual RCT	2 (3.3)
QE without control	13 (21.7)
QE with control	6 (10.0)
Prospective cohort	5 (8.3)
Retrospective cohort	1 (1.7)
None	24 (40.0)
IndePENDENT VARIABLE	
No Comparison	33 (0.55)
Intervention	13 (21.7)
Implementation strategy	14 (23.3)
Context	0
Implementation research theory or framework used^a^	
Determinants	36 (60.0)
Process	7 (11.7)
Evaluation	24 (40.0)
Implementation outcomes measured^a^	
Acceptability	27 (45.0)
Adoption	29 (48.3)
Appropriateness	16 (26.7)
Cost	16 (26.7)
Feasibility	16 (26.7)
Fidelity	26 (43.3)
Penetration	19 (31.7)
Sustainability	17 (28.3)
Health outcomes measured	25 (41.7)

*RCT* Randomized control trial, *QE* Quasi-experimental

^a^ ≥ 1 response per study possible

Nearly three-quarters of studies described empirical research (*n* = 43, 71.7%), the others being protocols for studies not yet completed (*n* = 17, 23.3%). Study populations included patients (*n* = 31, 51.7%), providers (*n* = 43, 71.7%), policymakers (*n* = 14, 23.3%), community members (*n* = 8, 13.3%), and researchers (*n* = 6, 10.0%); 34 (56.7%) studies included more than one of these study populations. Nineteen (31.7%) articles described formative implementation strategy design and nine (15.0%) described retrospective strategy specification. The majority (*n* = 36, 60%) of studies included an impact evaluation, the most common design being quasi-experimental with no control (*n* = 13, 21.7%) and a cluster randomized control trial (*n* = 9, 15.0%). Of the 17 studies that included a control, *n* = 9 tested the intervention and n = 7 tested the implementation strategy. Implementation frameworks were used by 47 (78.3%) studies, with 36 (60.0%) citing implementation determinant frameworks, 24 (40.0%) citing evaluation frameworks, and seven (11.7%) citing a process framework. A total of 44 (73.4%) studies evaluated implementation outcomes, most commonly adoption (*n* = 29), acceptability (*n* = 27), and fidelity (*n* = 26); 34 (56.7%) studies evaluated multiple implementation outcomes. Under half of studies (*n* = 25, 41.7%) evaluated health outcomes.

Across the 60 studies, 548 strategies were proposed, 282 (51.5%) of which were planned for future delivery and 266 (48.5%) had been delivered in the study. The total number of strategies described per study ranged from 1 to 46 (median = 6). Despite all 60 studies referencing the use of ERIC, just 32 (53.3%) studies explicitly matched specific implementation strategies used in the study to a specific ERIC strategy (Table 2). One study described strategy development being guided by other frameworks in addition to ERIC [47–49]; all other studies cited only ERIC as the guiding framework. The most commonly utilized ERIC strategies were (1) conduct educational meetings (*n* = 16, 2.9% of all 548 strategies proposed across studies); (2) audit and provide feedback (*n* = 15, 2.7%); (3) assess for readiness and identify barriers and facilitators (*n* = 13, 2.4%); and (4) build a coalition (*n* = 12, 2.2%). Of the 73 ERIC strategies, 9 (12.3%) were not cited at all. These include altering patient/consumer fees, changing liability laws, developing disincentives, making billing easier, preparing patients/consumers to be active participants, starting a dissemination organization, using capitated payments, using other payment schemes, and visiting other sites. Strategies from all ERIC strategy categories were described, though 5 of 9 (55.5%) strategies in the Utilize Financial Strategies category were not used.

**Table 2:** Descriptive statistics of implementation strategies

	Overall *N* = 548	Planned *n* = 282	Delivered *n* = 266
Implementation strategies	*n* (%)	*n* (%)	*n* (%)
Not matched to ERIC	153 (27.9)	71 (25.2)	82 (30.8)
Adapt and tailor to context			
Promote adaptability	5 (0.9)	4 (1.4)	1 (0.4)
Tailor strategies	8 (1.5)	4 (1.4)	4 (1.5)
Use data experts	2 (0.4)	1 (0.4)	1 (0.4)
Use data warehousing techniques	2 (0.4)	1 (0.4)	1 (0.4)
Change infrastructure			
Change accreditation or membership requirements	2 (0.4)	1 (0.4)	1 (0.4)
Change liability laws	0	0	0
Change physical structure and equipment	9 (1.6)	6 (2.1)	3 (1.1)
Change record systems	4 (0.7)	3 (1.1)	1 (0.4)
Change service sites	5 (0.9)	4 (1.4)	1 (0.4)
Create or change credentialing/licensure standards	1 (0.2)	1 (0.4)	0 (0.0)
Mandate change	3 (0.5)	2 (0.7)	1 (0.4)
Start a dissemination organization	0	0	0
Develop stakeholder interrelationships			
Build a coalition	12 (2.2)	5 (1.8)	7 (2.6)
Capture and share local knowledge	7 (1.3)	5 (1.8)	2 (0.8)
Conduct local consensus discussions	9 (1.6)	3 (1.1)	6 (2.3)
Develop academic partnerships	6 (1.1)	2 (0.7)	4 (1.5)
Develop an implementation glossary	1 (0.2)	1 (0.4)	0 (0.0)
Identify and prepare champions	11 (2.0)	8 (2.8)	3 (1.1)
Identify early adopters	1 (0.2)	1 (0.4)	0 (0.0)
Inform local opinion leaders	5 (0.9)	4 (1.4)	1 (0.4)
Involve executive boards	6 (1.1)	3 (1.1)	3 (1.1)
Model and simulate change	3 (0.5)	2 (0.7)	1 (0.4)
Obtain formal commitments	6 (1.1)	3 (1.1)	3 (1.1)
Organize clinician implementation team meetings	10 (1.8)	6 (2.1)	4 (1.5)
Promote network weaving	3 (0.5)	0 (0.0)	3 (1.1)
Recruit, designate, and train for leadership	5 (0.9)	4 (1.4)	1 (0.4)
Use advisory boards and workgroups	9 (1.6)	5 (1.8)	4 (1.5)
Use an implementation advisor	2 (0.4)	0 (0.0)	2 (0.8)
Visit other sites	0	0	0
Engage consumers			
Increase demand	3 (0.5)	2 (0.7)	1 (0.4)
Intervene with patients/consumers to enhance uptake and adherence	6 (1.1)	6 (2.1)	0 (0.0)
Involve patients/consumers and family members	3 (0.5)	1 (0.4)	2 (0.8)
Prepare patients/consumers to be active participants	0	0	0
Use mass media	3 (0.5)	1 (0.4)	2 (0.8)
Provide interactive assistance			
Centralize technical assistance	4 (0.7)	2 (0.7)	2 (0.8)
Facilitation	5 (0.9)	2 (0.7)	3 (1.1)
Provide clinical supervision	4 (0.7)	4 (1.4)	0 (0.0)
Provide local technical assistance	6 (1.1)	2 (0.7)	4 (1.5)
Support clinicians			
Create new clinical teams	1 (0.2)	1 (0.4)	0 (0.0)
Develop resource-sharing agreements	1 (0.2)	1 (0.4)	0 (0.0)
Facilitate relay of clinical data to providers	5 (0.9)	3 (1.1)	2 (0.8)
Remind clinicians	4 (0.7)	3 (1.1)	1 (0.4)
Revise professional roles	11 (2.0)	9 (3.2)	2 (0.8)
Train and educate stakeholders			
Conduct educational meetings	16 (2.9)	7 (2.5)	9 (3.4)
Conduct educational outreach visits	4 (0.7)	1 (0.4)	3 (1.1)
Conduct ongoing training	8 (1.5)	5 (1.8)	3 (1.1)
Create a learning collaborative	9 (1.6)	5 (1.8)	4 (1.5)
Develop educational materials	10 (1.8)	3 (1.1)	7 (2.6)
Distribute educational materials	5 (0.9)	2 (0.7)	3 (1.1)
Make training dynamic	6 (1.1)	4 (1.4)	2 (0.8)
Provide ongoing consultation	8 (1.5)	4 (1.4)	4 (1.5)
Shadow other experts	2 (0.4)	1 (0.4)	1 (0.4)
Use train-the-trainer strategies	3 (0.5)	3 (1.1)	0 (0.0)
Work with educational institutions	1 (0.2)	1 (0.4)	0 (0.0)
Use evaluative and iterative strategies			
Assess for readiness; identify barriers and facilitators	13 (2.4)	5 (1.8)	8 (3.0)
Audit and provide feedback	15 (2.7)	6 (2.1)	9 (3.4)
Conduct cyclical small tests of change	8 (1.5)	6 (2.1)	2 (0.8)
Conduct local needs assessment	11 (2.0)	7 (2.5)	4 (1.5)
Develop a formal implementation blueprint	11 (2.0)	8 (2.8)	3 (1.1)
Develop and implement tools for quality monitoring	4 (0.7)	3 (1.1)	1 (0.4)
Develop and organize quality monitoring systems	8 (1.5)	6 (2.1)	2 (0.8)
Obtain and use patient/consumer and family feedback	4 (0.7)	3 (1.1)	1 (0.4)
Purposely reexamine the implementation	5 (0.9)	3 (1.1)	2 (0.8)
Stage implementation scale-up	3 (0.5)	2 (0.7)	1 (0.4)
Utilize financial strategies			
Access new funding	3 (0.5)	3 (1.1)	0 (0.0)
Alter incentive/allowance structures	2 (0.9)	1 (0.7)	1 (1.2)
Alter patient/consumer fees	0	0	0
Develop disincentives	0	0	0
Fund and contract for the clinical innovation	1 (0.2)	0 (0.0)	1 (0.4)
Make billing easier	0	0	0
Place innovation on fee-for-service lists/formularies	1 (0.2)	1 (0.4)	0 (0.0)
Use capitated payments	0	0	0
Use other payment schemes	0	0	0

Eight (13.3%) studies did not include any component of strategy specification (i.e., they named strategies but did not describe them at all), representing 88 (16.1%) of the 548 strategies described across studies. Among the strategies that were specified (Table 3), the most common components described were action (*n* = 412, 75.2%), action target (*n* = 333, 60.8%), and actor (*n* = 314, 57.3%). The study team itself comprised the majority of actors specified for implementation strategies, accounting for 185 (58.9%) of the 314 actors specified; action targets were most commonly healthcare providers, accounting for 196 (58.9%) of 333 action targets specified. The least commonly specified components were fidelity (*n* = 17, 3.1%), adaptation (*n* = 100, 18.2%), action mechanism (*n* = 129, 23.5%), and targeted implementation outcomes (*n* = 148, 27.1%). Only 12 (20.0%) studies described adaptation of implementation strategies, of which two (3.3%) described the use of Implementation mapping and one the use of human-centered design (1.7%) to guide adaptation, while nine (15.0%) described more general stakeholder engagement without the use of a specific framework of the process. Only one (0.5%) strategy was tested for independent effects: Gachau et al. found that audit and feedback significantly improved 24 of 34 indicators of pediatric guideline adherence in Kenya [50].

**Table 3:** Descriptive statistics of implementation strategy specification

	Overall *N* = 548	Planned *n* = 282	Delivered *n* = 266
Dimension	*n* (%)	*n* (%)	*n* (%)
Actor^a^			
Unspecified (%)	234 (42.7)	178 (63.1)	56 (21.1)
Researcher/study team (%)	185 (33.8)	43 (15.2)	142 (53.4)
Provider (%)	91 (16.6)	42 (14.9)	49 (18.4)
Patient (%)	2 (0.4)	1 (0.4)	1 (0.4)
Policymaker (%)	30 (5.5)	10 (3.5)	20 (7.5)
NGO Staff (%)	2 (0.4)	1 (0.4)	1 (0.4)
Healthcare administration (%)	72 (13.1)	33 (11.7)	39 (14.7)
Community member (%)	26 (4.7)	3 (1.1)	23 (8.6)
Action target^a^			
Unspecified (%)	215 (39.2)	158 (56.0)	57 (21.4)
Non-human, e.g., systems, processes, agreements (%)	21 (3.8)	15 (5.3)	6 (2.3)
Researcher/Study Team (%)	26 (4.7)	5 (1.8)	21 (7.9)
Provider (%)	196 (35.8)	69 (24.5)	127 (47.7)
Patient (%)	47 (8.6)	24 (8.5)	23 (8.6)
Policymaker (%)	42 (7.7)	10 (3.5)	32 (12.0)
NGO Staff (%)	11 (2.0)	1 (0.4)	10 (3.8)
Healthcare administration (%)	67 (12.2)	14 (5.0)	53 (19.9)
Community member (%)	34 (6.2)	6 (2.1)	28 (10.5)
Specification of:			
Action (%)	412 (75.2)	171 (60.6)	241 (90.6)
Action Mechanism (%)	129 (23.5)	36 (12.8)	93 (35.0)
Temporality (%)	232 (42.3)	87 (30.9)	145 (54.5)
Dose (%)	177 (32.3)	69 (24.5)	108 (40.6)
Target Implementation Outcome(s) (%)	148 (27.1)	51 (18.1)	97 (36.6)
Justification (%)	157 (28.6)	112 (39.7)	45 (16.9)
Adaptation (%)	100 (18.2)	67 (23.8)	33 (12.4)
Fidelity (%)	17 (3.1)	1 (0.4)	16 (6.0)
Evidence of individual strategy effect (%)	1 (0.2)	0 (0.0)	1 (0.4)

^a^ ≥ 1 response per strategy possible

## Discussion

In the present systematic review, we found 60 studies that cited the use of ERIC strategies in LMIC settings. These studies included data from all WHO regions and focused on diverse health issues, with over 35% published in the final year of our review period, indicating a growing application of ERIC in LMICs. However, just over half explicitly matched implementation strategies employed to an ERIC strategy and 16% of strategies did not include any components of strategy specification. Moreover, a minority of studies employed randomized trials or high-quality quasi-experimental designs with controls, and just one study evaluated implementation strategy effectiveness.

While nearly half of the included studies did not explicitly match implementation strategies used in the study to a specific ERIC strategy, several notable points arose from the ERIC strategies that were reported. First, a wide variety of ERIC strategies were identified, with 88% of the 73 ERIC strategies represented. This suggests that almost all ERIC strategies can be applied to LMIC contexts. Yet, several strategies that seem critical to implementation in LMIC contexts—including capturing and sharing local knowledge, conducting local needs assessment, providing local technical assistance, and tailoring strategies—were not commonly cited in any of the included studies. This may be because these strategies were considered irrelevant or incompatible [35], redundant with other ERIC strategies [34], or part of routine processes for implementing health interventions [51–53] rather than discrete implementation strategies. Additionally, other ERIC strategies rarely, if ever, cited included those that required systems-level changes—such as change fees/incentives/billing, change policy, and enhancing local data systems and analysis—despite data suggesting organizational- and policy-level strategies are effective [54] and a critical component to closing the research-to-practice gap [55]. Finally, a number of strategies not used were those related to privatized health systems (e.g., alter consumer fees, change liability laws, use other payment schemes), which are less common in LMICs than centralized, public health systems, and thus may not be applicable or require adaptation in LMIC contexts. Further research is needed to explore why certain ERIC strategies have not been used in LMICs and if there are additional implementation strategies relevant to LMIC settings not currently included in the ERIC compilation.

Strengthening the evidence base for implementation strategies requires that their operationalization be reported in detail and that modifications to implementation strategies be systematically documented. While the included studies’ lack of ERIC strategy matching complicates the interpretation of specific ERIC strategy applicability to LMICs in this review, strategy matching itself may not be a requirement for understanding if and how certain implementation strategies are effective. However, implementation strategy components need to be specified in a way that allows their replication in research and practice and comparison across contexts [56, 57]. Among the studies reviewed here, all but eight included some type of strategy specification. However, most strategies only included specification of their action (75%), action target (61%), and actor (57%). Strategy details, such as temporality and dose, are necessary to replicate them in further testing. Moreover, mechanisms of action and fidelity, described for just 24% and 3% of strategies, respectively, are required for generating theory and selecting strategies that appropriately target contextual determinants [19]. Also concerning are how few strategies (18%) included a description of their adaptation process, which is likely necessary to meet the specific needs of the study context [2, 58–60]. Of the strategies that did include specification, most relied heavily on providers or research teams as actors and providers as action targets. This echoes our finding that few strategies addressing the organizational and policy levels were used and further highlights the dearth of research on implementation strategies that support population-level health improvements previously observed in high-income [61] and LMIC settings [39]. Moreover, the reliance on research team members as strategy actors threatens the generalizability of implementation strategies used in the studies to real-world settings.

Finally, though we had hoped to be able to assess ERIC strategy effectiveness in LMIC, just one strategy was evaluated in the studies included in our review. This is likely related to few studies defining strategy justification, mechanisms, and targeted implementation outcomes in their research. Instead, most studies looked at implementation outcomes without directly linking them to an identified determinant and a theory of change, precluding them from evaluating the individual strategy’s impact. Research teams may have lacked the resources to conduct a randomized control trial to rigorously test a strategy, as less than 20% of studies employed this design. However, while randomized control trials are the gold standard for research on intervention effectiveness, research on implementation strategies can successfully employ alternative designs, including interrupted time series, factorial, adaptive, and rollout designs [62, 63]. These designs can provide more flexibility and feasibility in resource-limited settings, while simultaneously maximizing external validity [64, 65].

The present study has several limitations. For one, we only focused on articles that used the ERIC compilation to inform study strategies and not more broadly defined implementation strategies. Therefore, our results may not be generalizable to all implementation strategy research in LMIC. We chose to focus on ERIC strategies as it is the taxonomy most commonly used in the field of implementation science, with over 3300 citations for the original [23] and refined [21] ERIC strategy papers and was developed using an expert review of existing strategy compilations and reviews. While we recognize that this biases toward research connected to non-LMIC academics, we note that around two-thirds of included studies were published in the final 2 years of our review period, suggesting that the use of ERIC is becoming more widespread in LMICs and highlighting the need to understand how to promote improved application and testing of these strategies. Second, we only included articles published in peer-reviewed journals. Owing to factors such as publication cost and language of these journals, this likely biased our findings to represent international and well-funded research. Further research is needed to explore non-ERIC strategy application in LMICs across languages and in the grey literature. Third, as we included study protocols in our sample, we cannot say with certainty that all strategies proposed will be applied in the research phase. However, the objective of this review was to provide a comprehensive description of the current state of this rapidly growing field (e.g., 35% of included papers published in the last year, 28% of studies still in the protocol phase), and inclusion of study protocols allows us to capture the most recent data. Finally, as many strategies employed were not matched to ERIC by the study authors, we were unable to draw conclusions about which may be the most relevant ERIC strategy(ies) in LMICs. However, those that did match indicated the individual ERIC strategies are applicable in LMICs.

## Conclusions

This systematic review demonstrated the broad and growing use of the ERIC strategy taxonomy in LMICs, with inconsistency in application and very limited testing of ERIC strategy effectiveness. Moving forward, we provide the following recommendations to promote the development of implementation strategies to more rapidly close the research-to-practice gap in LMICs. First, research in LMICs must move beyond merely describing strategies to evaluating their effects on implementation outcomes. Moreover, strategies that are tested need to be better specified so that their effectiveness may be compared across studies and contexts and their mechanisms of change can be understood. Researchers should also consider reporting how the strategy would be deployed under routine, non-research-related conditions as well to promote application beyond the study period. Finally, strategies targeting policy, organizational, and community-level determinants should be explored to encourage change that supports scale-up and sustainability of individual, research-based implementation efforts in LMICs. To catalyze these lines of research, there is a need for greater capacity-building among researchers in LMICs to gain training in implementation research. Moreover, this training should directly involve and/or emphasize methods for the engagement of diverse local stakeholders, such as policymakers and community members, who may be better situated to develop and implement strategies at the systems level. Research funders, governments, and other implementers should consider encouraging work that includes each of these components.

### Supplementary Information

**Additional file 1. **PRISMA 2020 checklist.

**Additional file 2. **ERIC LMIC search protocol 2023.07.15R2.

**Additional file 3. **Study descriptive information.

## Data Availability

All articles included in this systematic review are publicly available. The datasets used and/or analyzed during the current study are available from the corresponding author on reasonable request.

## Abbreviations

LMICs: Low- and middle-income countries
ERIC: Expert Recommendations for Implementing Change
WHO: World Health Organization
