# Supplementary material for: Application of the Expert Recommendations for Implementing Change (ERIC) compilation of strategies to health intervention implementation in low- and middle-income countries: a systematic review
Source: Implement Sci. 2023 Oct 30;18:56. doi: 10.1186/s13012-023-01310-2 (PMC10617067; doi:10.1186/s13012-023-01310-2)
Supplement: Supplementary file 2 — Additional file 2. ERIC LMIC search protocol 2023.07.15R2. [file 13012_2023_1310_MOESM2_ESM.docx]

**Use of the Expert Recommendations for Implementing Change (ERIC) compilation of implementation strategies in low- and middle-income countries**

Search Protocol

**Specific Aims**

1. Review the literature on the use of ERIC implementation strategies in low- and middle-income countries
   1. Which (ERIC) strategies have been used by investigators in LMIC settings?
      1. Use can include but is not limited to evaluation/c
   2. How were they used?
      1. Full specification of implementation strategy (i.e., actor, action, target, temporality, dose, implementation outcomes, justification)
      2. Target clinical intervention(s), health condition(s), and patient/client population(s)
   3. How were they adapted or changed?
   4. How were they chosen?
2. Assess evidence of the effectiveness of specific ERIC implementation strategies in low- and middle-income countries

**Methods**

***Search Strategy***

*Primary search: citations of Powell et al (2015) or citations of Waltz et al (2015) mentioning LMIC*

Web of Science:

TITLE: (A refined compilation of implementation strategies: results from the Expert Recommendations for Implementing Change (ERIC) project)

TITLE: (Use of concept mapping to characterize relationships among implementation strategies and assess their feasibility and importance: results from the Expert Recommendations for Implementing Change (ERIC) study)

TITLE: (A Compilation of Strategies for Implementing Clinical Innovations in Health and Mental Health)

THEN refine search using Countries/Regions. Exclude HICs.

<https://www.webofscience.com/wos/woscc/summary/1c541fcc-2c94-434b-a0bb-dc532d603f5a-00a8534b/relevance/1>

SCOPUS:

(REF(“A refined compilation of implementation strategies: results from the Expert Recommendations for Implementing Change (ERIC) project”) OR REF(“Use of concept mapping to characterize relationships among implementation strategies and assess their feasibility and importance: results from the Expert Recommendations for Implementing Change (ERIC) study”) OR REF(“A Compilation of Strategies for Implementing Clinical Innovations in Health and Mental Health”)) AND TITLE-ABS-KEY(Afghanistan OR Albania OR Algeria OR Samoa OR Angola OR Antigua OR Barbuda OR Argentina OR Armenia OR Azerbaijan OR Bangladesh OR Belarus OR Belize OR Benin OR Bhutan OR Bolivia OR Bosnia OR Herzegovina OR Botswana OR Brazil OR Bulgaria OR "Burkina Faso" OR Burundi OR Cambodia OR Cameroon OR "Cabo Verde" OR "Central African Republic" OR Chad OR Chile OR China OR Colombia OR Comoros OR Congo OR "Costa Rica" OR Ivoire OR Ivory OR Cuba OR Djibouti OR Dominica OR Dominican OR Ecuador OR Egypt OR Salvador OR Eritrea OR Ethiopia OR Fiji OR Gabon OR Gambia OR Georgia OR Ghana OR Grenada OR Guatemala OR Guinea OR Guinea-Bissau OR Guyana OR Haiti OR Honduras OR India OR Indonesia OR Iran OR Iraq OR Jamaica OR Jordan OR Kazakhstan OR Kenya OR Kiribati OR Korea OR Kosovo OR Kyrgyz OR Lao OR Laos OR Latvia OR Lebanon OR Lesotho OR Liberia OR Libya OR Lithuania OR Macedonia OR Madagascar OR Malawi OR Malaysia OR Maldives OR Mali OR Marshall OR Mauritania OR Mauritius OR Mexico OR Micronesia OR Moldova OR Mongolia OR Montenegro OR Morocco OR Mozambique OR Myanmar OR Namibia OR Nepal OR Nicaragua OR Niger OR Nigeria OR Pakistan OR Palau OR Panama OR "Papua New Guinea" OR Paraguay OR Peru OR Philippines OR Romania OR Russia OR Russian OR Rwanda OR Samoa OR "Sao Tome" OR Senegal OR Serbia OR Seychelles OR "Sierra Leone" OR "Solomon Islands" OR Somalia OR "South Africa" OR "Sri Lanka" OR "St. Lucia" OR "St. Vincent" OR Grenadines OR Sudan OR Suriname OR Swaziland OR Syrian OR Syria OR Tajikistan OR Tanzania OR Thailand OR Timor-Leste OR Togo OR Tonga OR Tunisia OR Turkey OR Turkmenistan OR Tuvalu OR Uganda OR Ukraine OR Uruguay OR Uzbekistan OR Vanuatu OR Venezuela OR Vietnam OR "West Bank" OR Gaza OR Yemen OR Zambia OR Zimbabwe OR Africa OR sub-saharan OR “developing country” OR “developing countries” OR “low-resource” OR “resource-limited” OR “resource-constrained” OR “low- and middle-income” OR LMIC OR “third world” OR “low-income country” OR “middle-income country”)

*Secondary search: papers mentioning “ERIC” and an LMIC*

PubMed:

(“Expert Recommendations for Implementing Change”[tw])

AND

(Afghanistan[tw] OR Albania[tw] OR Algeria[tw] OR Samoa[tw] OR Angola[tw] OR Antigua[tw] OR Barbuda[tw] OR Argentina[tw] OR Armenia[tw] OR Azerbaijan[tw] OR Bangladesh[tw] OR Belarus[tw] OR Belize[tw] OR Benin[tw] OR Bhutan[tw] OR Bolivia[tw] OR Bosnia[tw] OR Herzegovina[tw] OR Botswana[tw] OR Brazil[tw] OR Bulgaria[tw] OR Burkina Faso[tw] OR Burundi[tw] OR Cambodia[tw] OR Cameroon[tw] OR Cabo Verde[tw] OR Central African Republic[tw] OR Chad[tw] OR Chile[tw] OR China[tw] OR Colombia[tw] OR Comoros[tw] OR Congo[tw] OR Costa Rica[tw] OR Côte d'Ivoire[tw] OR Cote d’Ivoire[tw] OR Ivory[tw] OR Cuba[tw] OR Djibouti[tw] OR Dominica[tw] OR Dominican[tw] OR Ecuador[tw] OR Egypt[tw] OR Salvador[tw] OR Eritrea[tw] OR Ethiopia[tw] OR Fiji[tw] OR Gabon[tw] OR Gambia[tw] OR Georgia[tw] OR Ghana[tw] OR Grenada[tw] OR Guatemala[tw] OR Guinea[tw] OR Guinea-Bissau[tw] OR Guyana[tw] OR Haiti[tw] OR Honduras[tw] OR India[tw] OR Indonesia[tw] OR Iran[tw] OR Iraq[tw] OR Jamaica[tw] OR Jordan[tw] OR Kazakhstan[tw] OR Kenya[tw] OR Kiribati[tw] OR Korea [tw] OR Kosovo[tw] OR Kyrgyz [tw] OR Lao[tw] OR Laos[tw] OR Latvia[tw] OR Lebanon[tw] OR Lesotho[tw] OR Liberia[tw] OR Libya[tw] OR Lithuania[tw] OR Macedonia[tw] OR Madagascar[tw] OR Malawi[tw] OR Malaysia[tw] OR Maldives[tw] OR Mali[tw] OR Marshall[tw] OR Mauritania[tw] OR Mauritius[tw] OR Mexico[tw] OR Micronesia[tw] OR Moldova[tw] OR Mongolia[tw] OR Montenegro[tw] OR Morocco[tw] OR Mozambique[tw] OR Myanmar[tw] OR Namibia[tw] OR Nepal[tw] OR Nicaragua[tw] OR Niger[tw] OR Nigeria[tw] OR Pakistan[tw] OR Palau[tw] OR Panama[tw] OR Papua New Guinea[tw] OR Paraguay[tw] OR Peru[tw] OR Philippines[tw] OR Romania[tw] OR Russia[tw] OR Russian[tw] OR Rwanda[tw] OR Samoa[tw] OR Sao Tome[tw] OR Senegal[tw] OR Serbia[tw] OR Seychelles[tw] OR Sierra Leone[tw] OR Solomon Islands[tw] OR Somalia[tw] OR South Africa[tw] OR Sri Lanka[tw] OR St. Lucia[tw] OR St. Vincent[tw] OR Grenadines[tw] OR Sudan[tw] OR Suriname[tw] OR Swaziland[tw] OR Syrian[tw] OR Syria[tw] OR Tajikistan[tw] OR Tanzania[tw] OR Thailand[tw] OR Timor-Leste[tw] OR Togo[tw] OR Tonga[tw] OR Tunisia[tw] OR Turkey[tw] OR Turkmenistan[tw] OR Tuvalu[tw] OR Uganda[tw] OR Ukraine[tw] OR Uruguay[tw] OR Uzbekistan[tw] OR Vanuatu[tw] OR Venezuela[tw] OR Vietnam[tw] OR “West Bank”[tw] OR Gaza[tw] OR Yemen[tw] OR Zambia[tw] OR Zimbabwe [tw] OR developing countries[mh] OR “developing country”[tw] OR “developing countries”[tw] OR “low-resource”[tw] OR “resource-limited”[tw] OR “resource-constrained”[tw] OR “low- and middle-income”[tw] OR LMIC[tw] OR “third world”[tw] OR “low-income country”[tw] OR “middle-income country”[tw])

PsycINFO:

(“Expert Recommendations for Implementing Change”)

AND

(Afghanistan OR Albania OR Algeria OR Samoa OR Angola OR Antigua OR Barbuda OR Argentina OR Armenia OR Azerbaijan OR Bangladesh OR Belarus OR Belize OR Benin OR Bhutan OR Bolivia OR Bosnia OR Herzegovina OR Botswana OR Brazil OR Bulgaria OR Burkina Faso OR Burundi OR Cambodia OR Cameroon OR Cabo Verde OR Central African Republic OR Chad OR Chile OR China OR Colombia OR Comoros OR Congo OR Costa Rica OR Côte d'Ivoire OR Cote d’Ivoire OR Ivory OR Cuba OR Djibouti OR Dominica OR Dominican OR Ecuador OR Egypt OR Salvador OR Eritrea OR Ethiopia OR Fiji OR Gabon OR Gambia OR Georgia OR Ghana OR Grenada OR Guatemala OR Guinea OR Guinea-Bissau OR Guyana OR Haiti OR Honduras OR India OR Indonesia OR Iran OR Iraq OR Jamaica OR Jordan OR Kazakhstan OR Kenya OR Kiribati OR Korea OR Kosovo OR Kyrgyz OR Lao OR Laos OR Latvia OR Lebanon OR Lesotho OR Liberia OR Libya OR Lithuania OR Macedonia OR Madagascar OR Malawi OR Malaysia OR Maldives OR Mali OR Marshall OR Mauritania OR Mauritius OR Mexico OR Micronesia OR Moldova OR Mongolia OR Montenegro OR Morocco OR Mozambique OR Myanmar OR Namibia OR Nepal OR Nicaragua OR Niger OR Nigeria OR Pakistan OR Palau OR Panama OR Papua New Guinea OR Paraguay OR Peru OR Philippines OR Romania OR Russia OR Russian OR Rwanda OR Samoa OR Sao Tome OR Senegal OR Serbia OR Seychelles OR Sierra Leone OR Solomon Islands OR Somalia OR South Africa OR Sri Lanka OR St. Lucia OR St. Vincent OR Grenadines OR Sudan OR Suriname OR Swaziland OR Syrian OR Syria OR Tajikistan OR Tanzania OR Thailand OR Timor-Leste OR Togo OR Tonga OR Tunisia OR Turkey OR Turkmenistan OR Tuvalu OR Uganda OR Ukraine OR Uruguay OR Uzbekistan OR Vanuatu OR Venezuela OR Vietnam OR “West Bank” OR Gaza OR Yemen OR Zambia OR Zimbabwe OR Africa OR sub-saharan OR “developing country” OR “developing countries” OR “low-resource” OR “resource-limited” OR “resource-constrained” OR “low- and middle-income” OR LMIC OR “third world” OR “low-income country” OR “middle-income country”)

CINAHL:

(“Expert Recommendations for Implementing Change”)

AND

(Afghanistan OR Albania OR Algeria OR Samoa OR Angola OR Antigua OR Barbuda OR Argentina OR Armenia OR Azerbaijan OR Bangladesh OR Belarus OR Belize OR Benin OR Bhutan OR Bolivia OR Bosnia OR Herzegovina OR Botswana OR Brazil OR Bulgaria OR Burkina Faso OR Burundi OR Cambodia OR Cameroon OR Cabo Verde OR Central African Republic OR Chad OR Chile OR China OR Colombia OR Comoros OR Congo OR Costa Rica OR Côte d'Ivoire OR Cote d’Ivoire OR Ivory OR Cuba OR Djibouti OR Dominica OR Dominican OR Ecuador OR Egypt OR Salvador OR Eritrea OR Ethiopia OR Fiji OR Gabon OR Gambia OR Georgia OR Ghana OR Grenada OR Guatemala OR Guinea OR Guinea-Bissau OR Guyana OR Haiti OR Honduras OR India OR Indonesia OR Iran OR Iraq OR Jamaica OR Jordan OR Kazakhstan OR Kenya OR Kiribati OR Korea OR Kosovo OR Kyrgyz OR Lao OR Laos OR Latvia OR Lebanon OR Lesotho OR Liberia OR Libya OR Lithuania OR Macedonia OR Madagascar OR Malawi OR Malaysia OR Maldives OR Mali OR Marshall OR Mauritania OR Mauritius OR Mexico OR Micronesia OR Moldova OR Mongolia OR Montenegro OR Morocco OR Mozambique OR Myanmar OR Namibia OR Nepal OR Nicaragua OR Niger OR Nigeria OR Pakistan OR Palau OR Panama OR Papua New Guinea OR Paraguay OR Peru OR Philippines OR Romania OR Russia OR Russian OR Rwanda OR Samoa OR Sao Tome OR Senegal OR Serbia OR Seychelles OR Sierra Leone OR Solomon Islands OR Somalia OR South Africa OR Sri Lanka OR St. Lucia OR St. Vincent OR Grenadines OR Sudan OR Suriname OR Swaziland OR Syrian OR Syria OR Tajikistan OR Tanzania OR Thailand OR Timor-Leste OR Togo OR Tonga OR Tunisia OR Turkey OR Turkmenistan OR Tuvalu OR Uganda OR Ukraine OR Uruguay OR Uzbekistan OR Vanuatu OR Venezuela OR Vietnam OR “West Bank” OR Gaza OR Yemen OR Zambia OR Zimbabwe OR Africa OR sub-saharan OR “developing country” OR “developing countries” OR “low-resource” OR “resource-limited” OR “resource-constrained” OR “low- and middle-income” OR LMIC OR “third world” OR “low-income country” OR “middle-income country”)

EMBASE:

(“Expert Recommendations for Implementing Change”:ti,ab,tn,kw)

AND

(Afghanistan:ti,ab,tn,kw OR Albania:ti,ab,tn,kw OR Algeria:ti,ab,tn,kw OR Samoa:ti,ab,tn,kw OR Angola:ti,ab,tn,kw OR Antigua:ti,ab,tn,kw OR Barbuda:ti,ab,tn,kw OR Argentina:ti,ab,tn,kw OR Armenia:ti,ab,tn,kw OR Azerbaijan:ti,ab,tn,kw OR Bangladesh:ti,ab,tn,kw OR Belarus:ti,ab,tn,kw OR Belize:ti,ab,tn,kw OR Benin:ti,ab,tn,kw OR Bhutan:ti,ab,tn,kw OR Bolivia:ti,ab,tn,kw OR Bosnia:ti,ab,tn,kw OR Herzegovina:ti,ab,tn,kw OR Botswana:ti,ab,tn,kw OR Brazil:ti,ab,tn,kw OR Bulgaria:ti,ab,tn,kw OR “Burkina Faso”:ti,ab,tn,kw OR Burundi:ti,ab,tn,kw OR Cambodia:ti,ab,tn,kw OR Cameroon:ti,ab,tn,kw OR “Cabo Verde”:ti,ab,tn,kw OR “Central African Republic”:ti,ab,tn,kw OR Chad:ti,ab,tn,kw OR Chile:ti,ab,tn,kw OR China:ti,ab,tn,kw OR Colombia:ti,ab,tn,kw OR Comoros:ti,ab,tn,kw OR Congo:ti,ab,tn,kw OR “Costa Rica”:ti,ab,tn,kw OR Ivoire:ti,ab,tn,kw OR Ivory:ti,ab,tn,kw OR Cuba:ti,ab,tn,kw OR Djibouti:ti,ab,tn,kw OR Dominica:ti,ab,tn,kw OR Dominican:ti,ab,tn,kw OR Ecuador:ti,ab,tn,kw OR Egypt:ti,ab,tn,kw OR Salvador:ti,ab,tn,kw OR Eritrea:ti,ab,tn,kw OR Ethiopia:ti,ab,tn,kw OR Fiji:ti,ab,tn,kw OR Gabon:ti,ab,tn,kw OR Gambia:ti,ab,tn,kw OR Georgia:ti,ab,tn,kw OR Ghana:ti,ab,tn,kw OR Grenada:ti,ab,tn,kw OR Guatemala:ti,ab,tn,kw OR Guinea:ti,ab,tn,kw OR “Guinea-Bissau”:ti,ab,tn,kw OR Guyana:ti,ab,tn,kw OR Haiti:ti,ab,tn,kw OR Honduras:ti,ab,tn,kw OR India:ti,ab,tn,kw OR Indonesia:ti,ab,tn,kw OR Iran:ti,ab,tn,kw OR Iraq:ti,ab,tn,kw OR Jamaica:ti,ab,tn,kw OR Jordan:ti,ab,tn,kw OR Kazakhstan:ti,ab,tn,kw OR Kenya:ti,ab,tn,kw OR Kiribati:ti,ab,tn,kw OR Korea:ti,ab,tn,kw OR Kosovo:ti,ab,tn,kw OR Kyrgyz:ti,ab,tn,kw OR Lao:ti,ab,tn,kw OR Laos:ti,ab,tn,kw OR Latvia:ti,ab,tn,kw OR Lebanon:ti,ab,tn,kw OR Lesotho:ti,ab,tn,kw OR Liberia:ti,ab,tn,kw OR Libya:ti,ab,tn,kw OR Lithuania:ti,ab,tn,kw OR Macedonia:ti,ab,tn,kw OR Madagascar:ti,ab,tn,kw OR Malawi:ti,ab,tn,kw OR Malaysia:ti,ab,tn,kw OR Maldives:ti,ab,tn,kw OR Mali:ti,ab,tn,kw OR Marshall:ti,ab,tn,kw OR Mauritania:ti,ab,tn,kw OR Mauritius:ti,ab,tn,kw OR Mexico:ti,ab,tn,kw OR Micronesia:ti,ab,tn,kw OR Moldova:ti,ab,tn,kw OR Mongolia:ti,ab,tn,kw OR Montenegro:ti,ab,tn,kw OR Morocco:ti,ab,tn,kw OR Mozambique:ti,ab,tn,kw OR Myanmar:ti,ab,tn,kw OR Namibia:ti,ab,tn,kw OR Nepal:ti,ab,tn,kw OR Nicaragua:ti,ab,tn,kw OR Niger:ti,ab,tn,kw OR Nigeria:ti,ab,tn,kw OR Pakistan:ti,ab,tn,kw OR Palau:ti,ab,tn,kw OR Panama:ti,ab,tn,kw OR “Papua New Guinea”:ti,ab,tn,kw OR Paraguay:ti,ab,tn,kw OR Peru:ti,ab,tn,kw OR Philippines:ti,ab,tn,kw OR Romania:ti,ab,tn,kw OR Russia:ti,ab,tn,kw OR Russian:ti,ab,tn,kw OR Rwanda:ti,ab,tn,kw OR Samoa:ti,ab,tn,kw OR “Sao Tome”:ti,ab,tn,kw OR Senegal:ti,ab,tn,kw OR Serbia:ti,ab,tn,kw OR Seychelles:ti,ab,tn,kw OR “Sierra Leone”:ti,ab,tn,kw OR “Solomon Islands”:ti,ab,tn,kw OR Somalia:ti,ab,tn,kw OR “South Africa”:ti,ab,tn,kw OR “Sri Lanka”:ti,ab,tn,kw OR “St. Lucia”:ti,ab,tn,kw OR “St. Vincent”:ti,ab,tn,kw OR Grenadines:ti,ab,tn,kw OR Sudan:ti,ab,tn,kw OR Suriname:ti,ab,tn,kw OR Swaziland:ti,ab,tn,kw OR Syrian:ti,ab,tn,kw OR Syria:ti,ab,tn,kw OR Tajikistan:ti,ab,tn,kw OR Tanzania:ti,ab,tn,kw OR Thailand:ti,ab,tn,kw OR “Timor-Leste”:ti,ab,tn,kw OR Togo:ti,ab,tn,kw OR Tonga:ti,ab,tn,kw OR Tunisia:ti,ab,tn,kw OR Turkey:ti,ab,tn,kw OR Turkmenistan:ti,ab,tn,kw OR Tuvalu:ti,ab,tn,kw OR Uganda:ti,ab,tn,kw OR Ukraine:ti,ab,tn,kw OR Uruguay:ti,ab,tn,kw OR Uzbekistan:ti,ab,tn,kw OR Vanuatu:ti,ab,tn,kw OR Venezuela:ti,ab,tn,kw OR Vietnam:ti,ab,tn,kw OR “West Bank”:ti,ab,tn,kw OR Gaza:ti,ab,tn,kw OR Yemen:ti,ab,tn,kw OR Zambia:ti,ab,tn,kw OR Zimbabwe:ti,ab,tn,kw OR Africa:ti,ab,tn,kw OR “developing country”:ti,ab,tn,kw OR “developing countries”:ti,ab,tn,kw OR “resource-limited”:ti,ab,tn,kw OR “resource-constrained”:ti,ab,tn,kw OR “low- and middle-income”:ti,ab,tn,kw OR LMIC:ti,ab,tn,kw OR “third world”:ti,ab,tn,kw OR “low income country”:ti,ab,tn,kw OR “middle-income country”:ti,ab,tn,kw)

*Search Results* (7/12/2021):

| **Database** | **Number of results** | **Notes/Exclusions** |
| --- | --- | --- |
| Web of Science | 52 (Powell et al 2015)  12 (Waltz et al 2015) | None |
| SCOPUS | 61 | None |
| PubMed | 0 |  |
| PsycINFO | 0 | None |
| CINAHL | 1 (duplicate, not exported) | None |
| EMBASE | 2 | None |

*Search Results* (8/20/2021):

| **Database** | **Number of results** | **Notes/Exclusions** |
| --- | --- | --- |
| Web of Science | 56 (Powell et al 2015)  23 (Powell et al 2012)  11 (Waltz et al 2015) | None |
| SCOPUS | 87 | None |
| PubMed | 0 |  |
| PsycINFO | 0 | None |
| CINAHL | 1 (duplicate, not exported) | None |
| EMBASE | 2 | None |

*Search Results* (3/27/2023):

| **Database** | **Number of results** | **Notes/Exclusions** |
| --- | --- | --- |
| Web of Science | 121 (Powell et al 2015)  36 (Powell et al 2012)  22 (Waltz et al 2015) | None |
| SCOPUS | 154 | None |
| PubMed | 2 | None |
| PsycINFO | 1 | None |
| CINAHL | 4 | None |
| EMBASE | 7 | None |

Other sources:

- Reference lists of included articles
- “Cited by” lists of included articles

***Inclusion/Exclusion Criteria*:**

**Study participants:** Any

**Intervention target population:** Any

**Intervention of interest**: Any implementation strategy that is described/classified using the ERIC strategies (per ERIC)

**Primary outcome of interest:** Any/none

**Secondary outcomes:** Any/none

**Setting:** Any LMIC. World Bank criteria as of year of publication.

**Types of studies:** We will include any study design. These will include but are not limited to: formative/qualitative studies (e.g., contextual determinants and mapping to implementation strategies), case studies, pilot evaluations, uncontrolled evaluations, quasi-experimental evaluations, RCTs, economic evaluations, policy analyses. Systematic reviews, meta-analyses, study protocols, and commentaries are excluded.

**Additional limits, e.g. language, publication type:** No language limitations. Original research published in peer-reviewed journal. Published since Powell et al 2015.

***Study selection process*:**

Extract articles -

1. Search each database.
2. Note the number of results from each database.
3. Export the results to a common Covidence database and remove duplicates
4. Note the number of results with duplicates removed.

Review titles/abstracts of all articles -

1. Two reviewers screen every article in database for inclusion
   1. Screen title first
      1. If title indicates any potential for inclusion, read abstract
   2. Criteria for inclusion in the full-text review include: 1) reference to implementation of health-related intervention in title, abstract, or keywords; and 2) reference to LMIC in the title, abstract, and/or key words.
   3. If the reviewer is unsure about whether the article should be included in the full-text review, include a comment. These articles will be discussed by the group to decide on inclusion in the full-text review.
2. Reviewers meet regularly to discuss disagreements. If unable to come to consensus at title/abstract level, err on inclusion.
3. Note number of articles included after screening and number excluded.

Review of full text articles -

1. 2 reviewers independently download and read full text of articles included. Assess eligibility for inclusion.
   1. Studies must meet all criteria: 1) formative research and/or use of an ERIC implementation strategy, 2) in a LMIC setting
   2. For studies not meeting criteria above, note how they do not meet the criteria.
2. Note number of articles included.
3. Note number and reasons for articles excluded.

Data abstraction –

1. Develop data abstraction form which will include columns for author, date, setting, target health condition, target intervention, implementation strategy, implementation strategy specification, info on choice of implementation strategy and adaptation, study design, results, implementation outcomes, service delivery outcomes, health outcomes, other outcomes.
   1. Test the abstraction form with ~2 articles, revise the form, and then test with an additional ~2 articles.
2. Complete data extraction forms for articles to be included:
   1. One primary reviewer will be randomly assigned to each article, and they will abstract data for included article. One secondary reviewer will be randomly assigned to validate abstraction.

The data abstraction form will be reviewed for each article and discrepancies will be resolved by the group.
